# Automated seizure activity tracking and onset zone localization from scalp EEG using deep neural networks

**DOI:** 10.1371/journal.pone.0264537

**Published:** 2022-02-28

**Authors:** Jeff Craley, Christophe Jouny, Emily Johnson, David Hsu, Raheel Ahmed, Archana Venkataraman

**Affiliations:** 1 Department of Electrical and Computer Engineering, Johns Hopkins University, Baltimore, MD, United States of America; 2 School of Medicine, Johns Hopkins University, Baltimore, MD, United States of America; 3 Department of Neurology, University of Wisconsin Madison, Madison, WI, United States of America; 4 Department of Neurosurgery, University of Wisconsin Madison, Madison, WI, United States of America; McGill University, CANADA

## Abstract

We propose a novel neural network architecture, SZTrack, to detect and track the spatio-temporal propagation of seizure activity in multichannel EEG. SZTrack combines a convolutional neural network encoder operating on individual EEG channels with recurrent neural networks to capture the evolution of seizure activity. Our unique training strategy aggregates individual electrode level predictions for patient-level seizure detection and localization. We evaluate SZTrack on a clinical EEG dataset of 201 seizure recordings from 34 epilepsy patients acquired at the Johns Hopkins Hospital. Our network achieves similar seizure detection performance to state-of-the-art methods and provides valuable localization information that has not previously been demonstrated in the literature. We also show the cross-site generalization capabilities of SZTrack on a dataset of 53 seizure recordings from 14 epilepsy patients acquired at the University of Wisconsin Madison. SZTrack is able to determine the lobe and hemisphere of origin in nearly all of these new patients *without retraining the network*. To our knowledge, SZTrack is the first end-to-end seizure tracking network using scalp EEG.

## Introduction

Epilepsy is a chronic neurological disorder characterized by spontaneous and recurring seizures [1]. While often treated with medication, roughly 30% of epilepsy patients are *medically refractory* [2] and do not achieve seizure freedom with anti-epileptic drugs [2]. Alternative treatments for these patients rely on our ability to detect, track, and localize seizure activity in their brains. Namely, if we can determine that the seizures originate from a discrete seizure onset zone (SOZ), then the most effective treatment is to surgically remove this region [3]. Multichannel electroencephalography (EEG) is the first modality used in the clinical evaluation of epilepsy. At present, the EEG is *visually scanned* for electrographic signatures of a seizure. This process is time consuming, requires specialized expertise, and is prone to human error [4].

Automated methods for scalp EEG have largely focused on the simpler problem of seizure detection. Here, the EEG signals are first windowed into short epochs, from which a set of features are extracted. Next, a classifier is trained on these features to declare each epoch as “seizure” or “baseline” [5]. The field has explored several features that capture *ictal*, i.e., seizure related, morphologies in the data. These features include cross-channel correlation [6], spectral power [7, 8], approximate entropy [9], Lyapunov exponents [10], and wavelet coefficients [11]. While informative, these features are not robust to patient heterogeneity [12] and the high-amplitude artifacts present in EEG data. As a result, traditional seizure detectors were trained and evaluated on a patient-specific basis [6, 7, 13], which is impractical during a prospective clinical review.

The rise of deep learning has prompted a new direction for seizure detection via neural networks. In the simplest case, hand-crafted feature extraction is combined with multi-layer perceptrons to classify short windows of the EEG segment [14]. More advanced methods have used 2D Convolutional Neural Networks (CNNs) to detect seizures based on EEG spectrograms [15, 16] and 1D CNNs to learn discriminative features directly from the EEG time series [17–19]. In parallel to the window-wise encoding, time-series data are often modeled via Recurrent Neural Networks (RNNs). Broadly, RNNs track temporal relationships by passing hidden states between connected network components. RNNs may be applied to either the raw EEG signal itself [20], or to features extracted from windows of the original EEG [21]. RNNs can also be coupled with CNN architectures, as in the work of [22]. Here, the CNN encodings extracted from longer (i.e., 101 second) clips of EEG were classified using an RNN network. Extending the combined CNN/RNN approach, the work of [23] couples a 1D CNN with an RNN to classify seizure activity on a shorter one-second timescale.

In recent years, Graph Convolutional Networks (GCNs) have become popular for multichannel analysis of EEG data. Broadly, GCNs extend the traditional convolutional architectures, which operate on a regular grid, to arbitrary graphs [24, 25]. Citing the network structure of the brain, GCN approaches in epilepsy encode the underlying connectivity of the brain, for example through spatial proximity or diffusion MRI pathways, directly into the filtering operations of the network. In the work of [26], spectral features derived from the Fast Fourier Transform are analyzed using GCNs to classify 10 second windows of multi-channel EEG as either containing or not containing seizure activity. Along the same lines, the work of [27] uses temporal GCNs to detect the presence of seizure activity in long (96 second) sequences. Going one step further, the authors of [28] learn subject-specific graphs for (temporal) seizure prediction using intracranial EEG. Finally, GCNs have appeared in conjunction with RNNs in other fields. For example, the work of [29] used this combination to the problem of emotion recognition from EEG, and the work of [30] apply a spatio-temporal GCN [31] to a Brain-Computer Interface (BCI) motor imagery task.

While deep networks achieve higher accuracies than purely model-based techniques [23]. simply detecting the onset and offset times of a seizure has limited translational value. Rather, clinicians must understand the *manifestation* of seizure activity to plan downstream therapeutics. Automated seizure localization from scalp EEG has received comparatively less attention and has largely focused on improving the EEG spatial resolution (typically 20–40 sensors) by deconvolving the signals into current dipoles [32, 33] or distributed sources [34–36] at the millimeter scale. However, these *inverse solvers* are sensitive to physiological noise, the number of EEG channels, and the underlying head model [37, 38]. With that said, a recent prospective study [39] suggested that EEG source localization information can be used to alter presurgical evaluation and in many cases shows a high degree of concurrence with MRI-based methods. However, the authors note that source has yet to be widely adopted in the clinic due to difficulties in its interpretation and the lack of robust automated methods.

Thus, we take an alternative approach and combine the problems of detection and localization to identify the onset and propagation of electrode-level seizure activity in clinical EEG data. In this paper, we introduce SZTrack, the first end-to-end network for multichannel seizure activity tracking. SZTrack uses a combined convolutional and recurrent approach to perform classification of seizure activity in individual EEG electrodes at timescales of 1 second, thus generating predictive maps of seizure activity at each time-step. While the architecture operates on each EEG electrode individually, we propose two novel aggregation techniques during training to leverage multichannel phenomena in the EEG data. Our first aggregation technique is to pool the channel-wise classifications into a single patient-wise seizure detection. This strategy allows us to train the network using standard clinical annotations of the seizure onset and offset. It also accommodates the fact that seizure activity may be present in only a subset of the EEG electrodes at a given time. Our second aggregation technique is to combine the *onset information* across channels inanterior vs. posterior head regions andright vs. left hemispheres into a single SOZ prediction. Once again, this strategy allows us to train our channel-wise architecture based on coarse SOZ annotations provided during clinical review. We evaluate SZTrack on two clinical EEG datasets acquired at the Johns Hopkins Hospital and the University of Wisconsin Madison. We demonstrate that SZTrack achieves comparable seizure detection performance to state-of-the-art deep learning approaches. In addition, it can reliably localize the SOZ in a leave-on-patient-out cross validation setting. Finally, SZTrack shows promising cross-site generalization between the two datasets, which provides further evidence of its clinical utility.

## SZTrack: An end-to-end seizure tracking model

**Fig 1:**
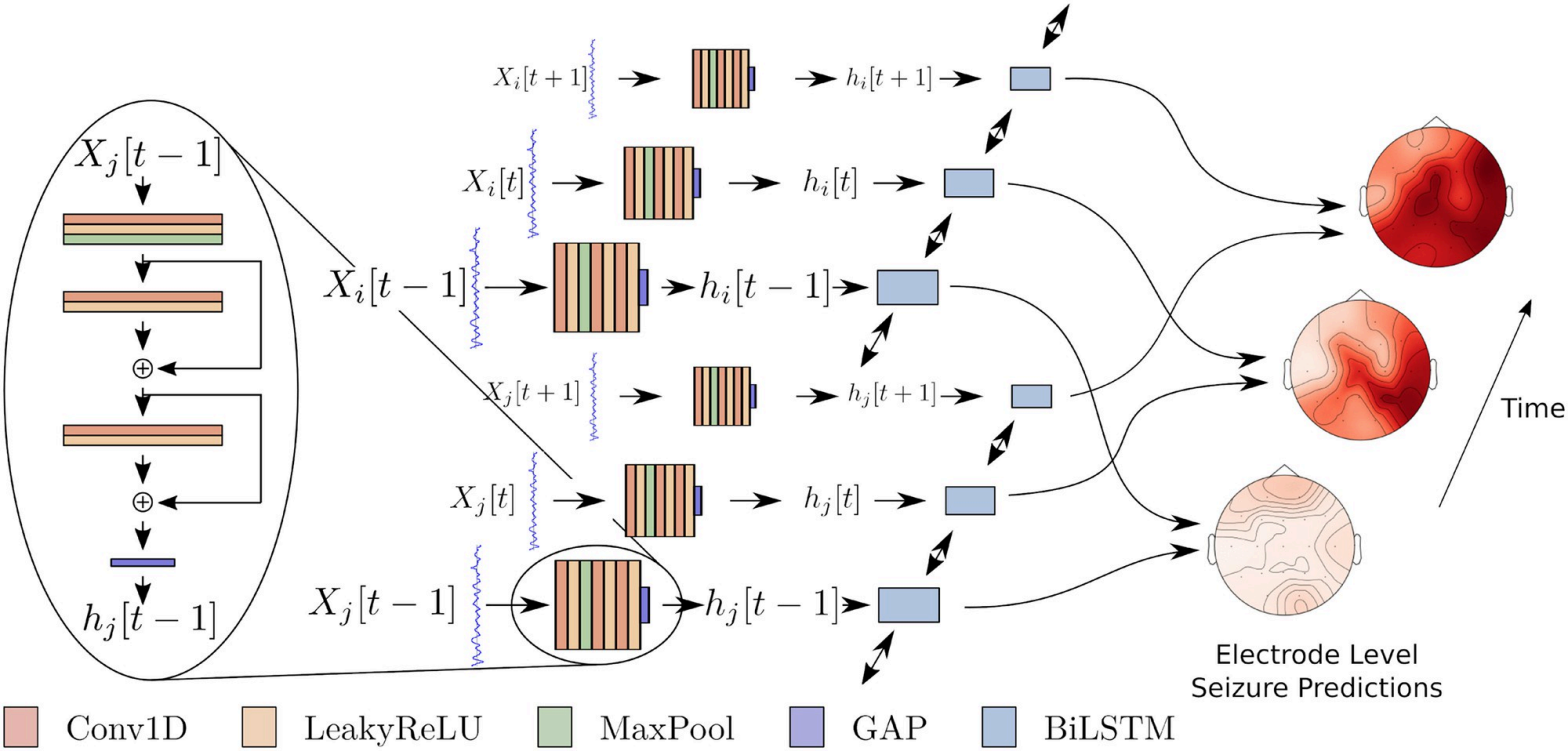
SZTrack architecture. Individual EEG electrode signals are fed through a 1D CNN (left). The sequences of representations are fed through the BLSTM layer and then classified for seizure activity in each electrode.

Fig 1 illustrates our SZTrack architecture. We first extract a hidden representation at the electrode level by applying a 1D CNN encoder to each one-second window of the time series (left). The encoding sequence for each electrode is passed through a Bidirectional Long Short-Term Memory (BLSTM) unit to determine channel-wise seizure activity. As indicated in Fig 1, the CNN and BLSTM parameters are shared across EEG channels, thus providing a compact deep network architecture. The following subsections describe the SZTrack components as well as the aggregation strategy used to train SZTrack for electrode level predictions from seizure onset, offset, and coarse localizations. All models were implemented in PyTorch 1.5.1. Our code is publicly available for download at https://engineering.jhu.edu/nsa/links/.

### CNN encoding to capture instantaneous phenomena

The CNN encoder (Fig 1, left) extracts feature representations directly from one-second windows of each EEG electrode signal. Let *X*_*i*_[*t*] be the signal in EEG electrode *i* during window *t* and **X** represent the combined signals for the entire recording. The signal *X*_*i*_[*t*] is passed through a 3 layer 1D CNN encoder to generate a hidden representation *h*_*i*_[*t*]. The first layer consists of 20 kernels (length = 7 samples and padding = 3 samples), followed by a LeakyReLU nonlinearity [40], Max Pooling (kernel = 2 samples) and Batch Normalization [41]. The second and third layers use 20 kernels (length = 3 samples and padding = 1 sample), followed by a LeakyReLU nonlinearity and Batch Normalization. Residual connections are added in the second and third layers to ensure a smooth flow of gradient information [42]. Finally, we apply global average pooling resulting in a length 20 hidden representation *h*_*i*_[*t*] for each electrode. CNN parameters are shared for all electrodes to ensure a consistent feature representation.

### Seizure tracking via recurrent neural networks

The working hypothesis in focal epilepsy is that a seizure originates from a discrete SOZ and spreads over time to involve other areas of the brain [43]. This spreading pattern is unique across patients, occuring at different time scales and encompassing different spatial extents [44]. We capture this temporal evolution using a BLSTM (Fig 1, right), which captures both long-term and short-term dependencies [45]. Formally, the CNN encodings *h*_*i*_[*t*] are passed through a BLSTM layer of 40 hidden units. This comparatively large size provides the representational flexibility to track the seizure evolution on longer (i.e., minute-level) time scales. We have tied the BLSTM weights across the electrodes to prevent SZTrack from biasing its predictions towards certain areas of the scalp. Let *o*_*i*_[*t*] be the output of the BLSTM for electrode *i* at time *t*. The channel-wise seizure prediction *Y*_*i*_[*t*] in EEG electrode *i* and time window *t* is made via a simple softmax assignment, i.e., $P\left({\hat{Y}}_{i}\left[t\right]|X\right)=\text{softmax}\left(W^{T}o_{i}\left[t\right]+b\right)$.

### Max pooling for global seizure prediction

Although SZTrack is designed to *track* the temporal evolution of seizure activity, we only have access to coarse seizure onset and offset times for training. Therefore, we develop a max-pooling strategy to aggregate the electrode level predictions ${\hat{Y}}_{i}\left[t\right]$ into recording-level predictions $\hat{Y}\left[t\right]$ for each one-second window *t*. Formally, the global prediction *Y*[*t*] at window *t* is predicted as the maximum predicted probability of seizure in any individual channel, i.e.,

$$\begin{array}{c} P\left(\hat{Y}\left[t\right]=1|X\right)=\underset{i}{\text{max}}P\left({\hat{Y}}_{i}\left[t\right]=1|X\right). \end{array}$$ (1)

Effectively, when one channel enters the seizure state, the network registers a seizure, accounting for the fact that the activity may concentrate in a single electrode or subset of electrodes. This flexibility allows SZTrack to learn seizure spreading patterns at the electrode resolution with only onset/offset training labels.

### Lateralization and anterior vs. posterior classification

**Fig 2:**
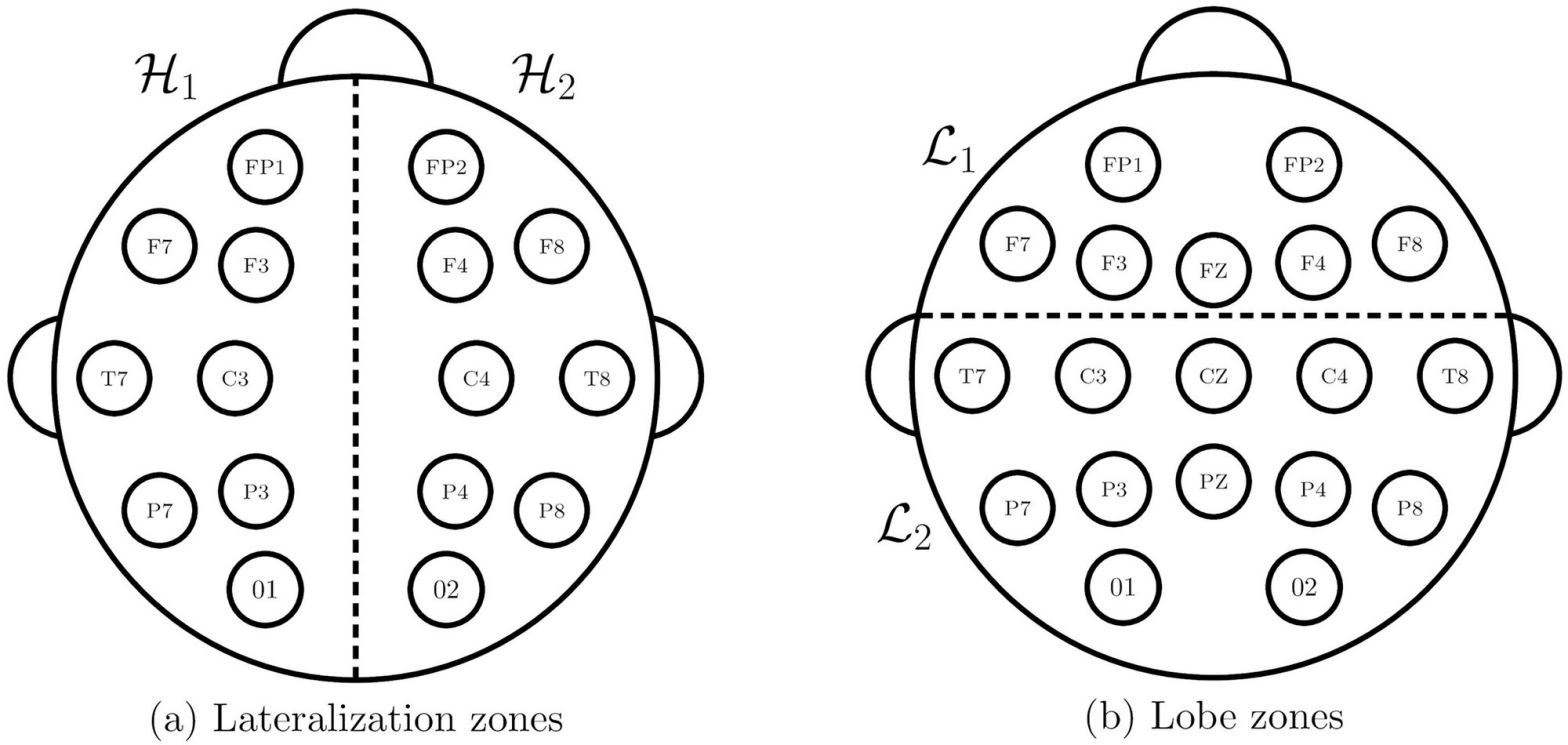
Localization zones and electrode connectivity graph. Partition of EEG electrodes into zones to train our network based on coarse hemisphere (a) andanterior and posterior head regions (b).

Similar to the coarse temporal information, our EEG datasets contain only hemisphere and lobe annotations of the seizure onset, such as “left frontal” or “right temporal”. Thus, in order to train SZTrack with these labels, we aggregate electrode level seizure predictions ${\hat{Y}}_{i}\left[t\right]$ according to the two partitions illustrated in Fig 2(a) and 2(b). In one partition, Fig 2(a), the EEG electrodes are divided into the left and right hemispheres, denoted $H_{1}$ and $H_{2}$, respectively. In the other partition, Fig 2(b), the EEG electrodes are divided intoanterior and posterior head regions, denoted $L_{1}$ and $L_{2}$, respectively. This classification boundary is defined such that the anterior head region coarsely aligns with frontal lobe seizure foci, while posterior head region contains temporal and parietal foci.

To arrive at a hemisphere oranterior and posterior prediction, we combine first order differences in *P*(*Y*[*t*] = 1 ∣ **X**), denoting seizure onset times, with electrode level predictions. Let Δ*P*_*on*_[*t*] capture the transition from baseline to seizure at time *t*. Mathematically,

$$\begin{array}{c} \Delta P_{on}\left[t\right]=\text{max}(P(\hat{Y}\left[t\right]=1|X)-P(\hat{Y}\left[t-1\right]=1|X),0) \end{array}$$ (2)

As reported in Eq (2), Δ*P*_*on*_[*t*] will approach 1 for confident transitions into seizure, and will be 0 if predicted seizures stays the same or decreases. These differences Δ*P*_*on*_[*t*] are multiplied by the seizure activity at time *t*, *P*(*Y*_*i*_[*t*]∣**X**), summed, and normalized to create a channel-level SOZ onset predictions *P*(*L*_*i*_ = 1∣*X*).

$$\begin{array}{c} P\left(L_{i}=1|X\right)=\frac{\sum_{t=0}^{T-2}\Delta P_{on}\left[t\right]P\left({\hat{Y}}_{i}\left[t\right]=1|X\right)}{\sum_{i=1}^{M}\sum_{t=0}^{T-2}\Delta P_{on}\left[t\right]P\left({\hat{Y}}_{i}\left[t\right]=1|X\right)} \end{array}$$ (3)

Effectively, Eq (3) computes the predicted seizure activity in channel *i* at time *t*, $P({\hat{Y}}_{i}\left[t\right]=1|X)$, weighted by the onset activity Δ*P*_*on*_[*t*] at that time. Notice that this aggregation relies on both accurate temporal onset detection via Δ*P*_*on*_[*t*] and spatial electrode prediction via *P*(*Y*_*i*_ = 1∣**X**) for a correct localization result. These predictions represent the posterior probability map of the SOZ electrode.

During training, the electrode onset scores *P*(*L*_*i*_ = 1∣**X**) are aggregated according to the regions defined in Fig 2(a) and 2(b) to create region level onset scores, $\hat{h}=P\left(\text{Hemi}=j|X\right)=\sum_{i\in H_{j}}\left(L_{i}=1|X\right)$ and $\hat{l}=P\left(\text{Region}=j|X\right)=\sum_{i\in L_{j}}\left(L_{i}=1|X\right)$. These hemisphere andanterior and posterior predictions are trained using cross-entropy loss function using true labels *h* and *l*, thus allowing SZTrack to learn electrode-level patterns from coarse clinical annotations.

### Validation strategy

We evaluate the seizure detection and localization performancesin separate experiments using leave-one-patient-out cross validation (LOPO-CV). Thiscross validation strategy mimics a standard clinical review by quantifying how well each method generalizes to unseen patients. In the detection experiment, we consider the temporal overlap between clinically provided seizure labels and the seizure predictions of SZTrack. In the localization experiment, onset weight in predicted by SZTrack in each the localization based divisions in Fig 2 is considered. Our detection and localization experiments are further detailed in the following subsections.

#### Seizure onset/offset detection

We train SZTrack using a cross-entropy loss between the recording-level seizure prediction $P(\hat{Y}\left[t\right]=1|X\left[t\right])$ and the clinician annotation of whether not a seizure is occurring at time window *t*. To mitigate over-fitting, training is done for 50 epochs with a weight decay of 0.0001 and a batch size of 4. The learning rate is set at 0.01 and reduced by a factor of 0.5 every 20 epochs.

Performance is evaluated at the one-second window level and by correct classification of the seizure period within each EEG recording. We adopt the strategy of [23], in which the detection threshold is calibrated during each LOPO-CV fold to allow 2 minutes of false positive detection per hour on the training data. At the window level, we report sensitivity, specificity, Area Under the Receiver Operating Characteristc (AU-ROC), and Area Under the Precision-Recall curve (AU-PR) without assuming any temporal dependencies. At the seizure level, we first identify continuous intervals that cross the calibrated detection threshold as “predicted seizures”.

The end of a seizure interval is typically corrupted by high levels of artifact (e.g., muscle and eye movements from lingering spasms). Thus, seizure offsets are clinically more difficult to identify, and post-seizure EEG is often mis-classified as a continuation of seizure activity. Since clinical evaluation of epilepsy focuses on the seizure onset and evolution behavior, we adopt a strategy that rewards true seizure detection (i.e., maximizing sensitivity) without penalizing the model for continuing post-seizure predictions. Predicted seizure intervals that overlap with annotated seizure activity are considered true positives, while intervals occurring exclusively during baseline are considered false positives. We note that this quantification strategy has been used previously in the seizure detection literature in [46] Predicted seizure intervals that overlap with annotated seizure activity are considered true positives, while intervals occurring exclusively during baseline are considered false positives. We report the seizure level metrics False Positive Rate (FPR), computed as the number of false positives per hour, and sensitivity, computed as the ratio of accurately classified seizures to missed seizures. We also report average latency for true positive detections. These metrics quantify the clinically relevant need for accurate seizure detection (sensitivity) with low latency and a small number of false positive detections.

#### Seizure localization

We evaluate lobe and lateralization accuracy in separate experiments on recordings clipped from 15 seconds prior to 30 seconds after seizure onset. This clipping mitigates the influence of physiological confounds, such as eye and muscle movements, in the challenging localization task. In each case, the loss is a weighted combination of the cross-entropy seizure detection loss:

$$\begin{array}{c} \begin{array}{cc} & L=\lambda_{sz}CE_{sz}\left(\hat{y},y\right)+CE_{hemi}\left(\hat{h},h\right),\;\text{or}\\ & L=\lambda_{sz}CE_{sz}\left(\hat{y},y\right)+CE_{region}\left(\hat{l},l\right) \end{array} \end{array}$$ (4)

We initialize SZTrack via the models learned in the corresponding LOPO-CV seizure detection experiment and retrain the network for 50 epochs with the combined loss function in Eq (4) using the same weight decay and learning rate schedule described above. To quantify robustness, we investigate the LOPO-CV performance while sweeping the detection loss weight λ_*sz*_ from (0.1 − 1.0).

#### Baseline models

**Fig 3:**
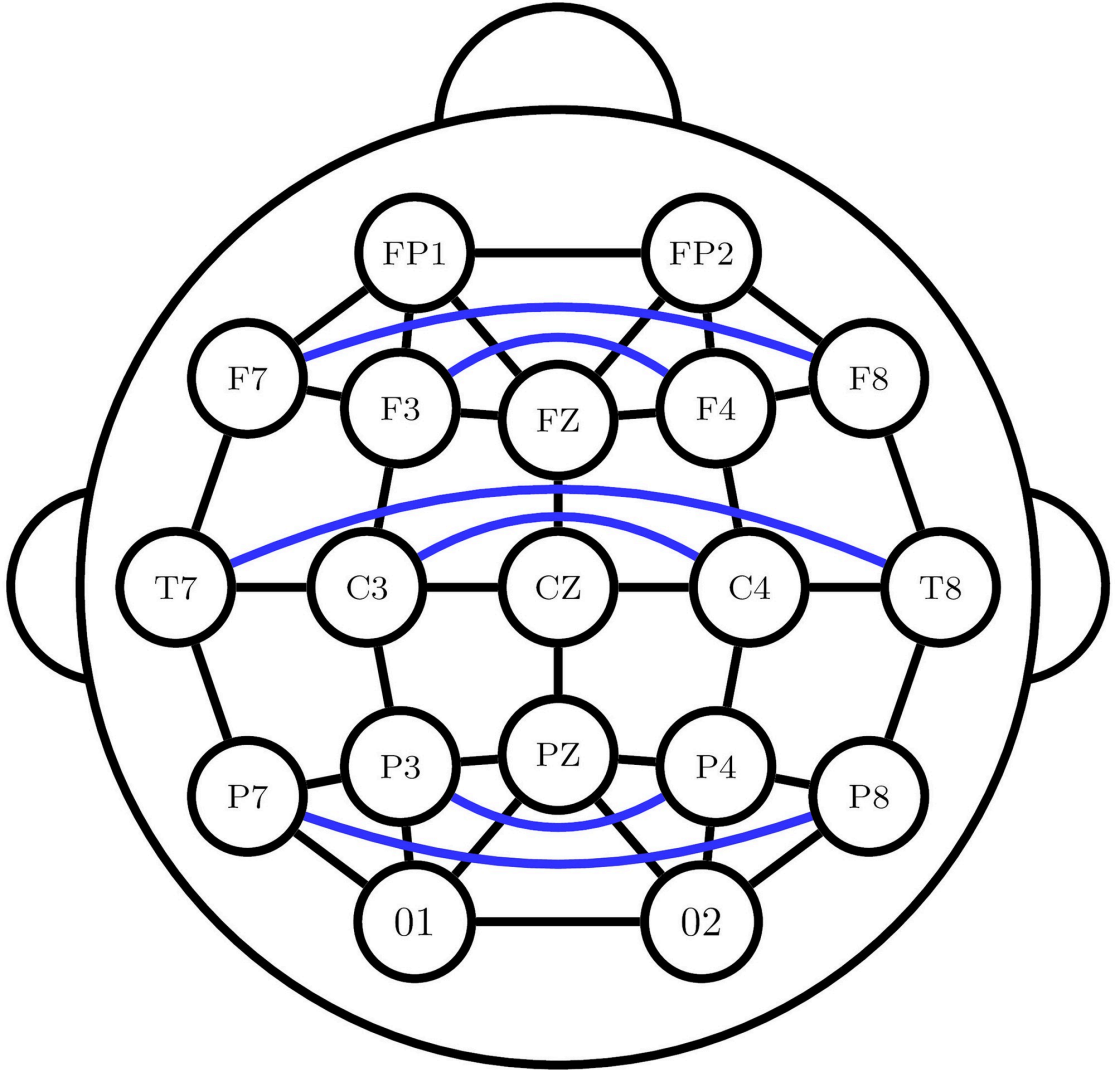
Electrode connectivity graph. Electrode connectivity graph used in GCN baselines.

We compare SZTrack to an ablated model, in which we remove the BLSTM layer. This model, which we call No-BLSTM, allows us to evaluate the benefit of tracking temporal dependencies in the EEG data. We also adapt to recently published deep learning models for seizure detection that use GCNs for spatial information fusion. In both cases, we use the graph defined in Fig 3, which connects neighboring and contralateral EEG electrodes.

The first model is a temporal GCN (TGCN) introduced in [27]. We rely on “Architecture II” from the paper, which achieves the best performance across most of the evaluation metrics in the original work. For comparison with SZTrack, we remove the max pooling along the temporal dimension and the average pooling along the spatial dimension, which allows the TGCN to output predictions at the electrode-level and one-second window-level resolution. The second and third GCN networks are introduced in [26] and apply the propagation rule in [47] along with graph pooling for sequence detection. Here, the Shallow-GCN model uses two GCN layers with 64 and 128 hidden units, respectively, followed by a single linear classification layer. The Deep-GCN model uses five GCN layers with increasing hidden sizes of 16, 16, 32, 64, and finally 128, followed by two linear layers with 30 and 20 hidden units before a final classification layer. Once again, we adapt the networks by removing the graph pooling layer to allow for electrode-level predictions. Unlike the previous methods, the Shallow-GCN and Deep-GCN operate on spectral input features. We construct this 10-dimensional input by extracting the spectral power in 10 equally-spaced frequency.

Finally we compare SZTrack against two multichannel CNN baselines and a multichannel CNN-BLSTM baseline that have recently appeared in the seizure detection literature. These models differ from SZTrack in that they *output a single global seizure prediction at every time window*. Hence, these models are *incapable of tracking seizure activity* at the resolution of individual electrodes, which is the goal of SZTrack. The Wei-CNN baseline [17] uses 5 CNN layers followed by 2 linear layers. The CNN-2D baseline operates on the short-time Fourier transform images. Four CNN layers are applied before a final linear layer is applied for classification. The CNN-BLSTM presented in [23] extracts features from the multichannel EEG signal using a CNN before classifying the sequence of features using a BLSTM layer.

We include all models in the our detection experiments. Empirically, all baselines except the CNN-BLSTM output noisy seizure detections, since they are made independently for each one-second window. Therefore, we smooth the predictions of these baseline models by averaging the outputs over 20 seconds. This smoothing procedure is omitted for SZTrack and the CNN-BLSTM. We assess the localization performance for SZTrack and the No-BLSTM and TCGN baselines. We omit the multichannel architectures, as they cannot output localization information at the window level. Similarly, we omit the Shallow-GCN and Deep-GCN baselines, as they do not include a temporal modeling component to capture seizure onset and evolution. In addition, we adapt the CNN-BLSTM models from our detection experiment to localization by adding a linear classification layer operating on the final hidden state of the BLSTM. We re-train these CNN-BLSTM models using the detection models as a starting point for an additional localization baseline. These models are trained explicitly for localization and λ_*sz*_ is set to 0 accordingly.

## Results

### Clinical EEG datasets

*JHH Dataset:* Our primary EEG dataset consists of 201 seizure recordings obtained from 34 focal epilepsy patients undergoing presurgical evaluation in the Johns Hopkins Hospital between 2016–2019. Patients ranged from 6–77 years with a mean age of 35.7 ± 16.8 years. The dataset contains 18 females and 16 males. Inclusion criteria were that the patient was a candidate for a focal resection with planned intracranial monitoring in the future. As part of this criteria, all patients had a well-characterized seizure onset zone based on the available clinical data. Exclusion criteria included non-epileptic seizures, generalized epilepsy, and patients who were not deemed to be surgical candidates. Many patients in the dataset separately underwent MRI or PET imaging. These scans revealed a range of structural abnormalities, including but not limited to focal cortical dysplasia (FCD), mesial temporal sclerosis (MTS), white matter disease, encephalocele, and gliomas. Table 1 summarizes the patient characteristics. Where available, we have included imaging notes from the patient medical record.

**Table 1:** Patient demographics and clinical attributes for our JHH evaluation dataset (N = 34) and UWM generalization dataset (N = 15).

	JHH Dataset	UWM Dataset
Seizure Type	Focal Epilepsy	Pediatric Focal Epilepsy
Has localizations?	Yes	Yes
Number of patients	34	15
Average Age	35 ± 16 years	12.8 ± 3.1 years
Minimum/Maximum Age	6/77 years	8/17 years
Number of Males/Females	16/18	10/5
Seizures per Patient	5.9 ± 5.8	6.6 ± 7.9
Minimum/Maximum Seizures per Patient	1/24	1/33
Average EEG per Patient	1.8 ± 1.8 hours	2.1 ± 0.9 hours
Average Seizure Duration	112 seconds	60 seconds
Minimum/Maximum Seizure Duration	13/979 seconds	13/212 seconds
Indicated Conditions	Cavernoma, MTS, perventricular heterotopia, stroke, encephalocele, FCD, low grade glioma	Focal cortical dysplasia, gliosis, encephalitis, encephalocele
Lesional/Non-Lesional/Unspecified	24/3/7	14/0/1
Temporal/Extra-Temporal	26/8	3, 12
Posterior/Anterior	27/7	10/5
Right/Left	18/16	6/9

All EEG were recorded on a Nihon Kohden system with a built-in amplifier. A 60 Hz notch filter, 70 Hz low pass filter, and 0.016 high pass filter were applied to the data after acquisition. The data was recorded at 200 Hz using the 10–20 electrode placement system [47]. For our analysis, the EEG recordings have been clipped to include roughly 10 minutes of pre-seizure and post-seizure activity.

*UWM Dataset:* Our generalization dataset consists of 53 seizure recordings from 15 pediatric patients admitted to University of Wisconsin-Madison (UWM) from February 2018 to December 2019. Patients ranged from 8–17 years with a mean age of 13 ± 3.1 years. The dataset contains 5 females and 10 males. Inclusion criteria included a suspected focal onset of the epileptic seizures, as characterized by expert review of the medical record. As the UWM dataset was drawn from a larger study of multimodal neuroimaging, inclusion criteria also included that the patient underwent MRI scanning with available T1 MRI and resting-state fMRI. However, the imaging data was not used in the present study. Of the 19 patients at UWM that met the inclusion criteria, 4 patients were excluded, 1 due to prior resection, 1 patient whose EEG recordings contained only auras, and 2 patients with indeterminate seizure onset zone. Similar to the JHH dataset, many patients had structural brain abnormalities visible on MRI and PET, such as FCD, encephalocele, gliosis, MTS, and encephalitis. Table 1 summarizes the patient characteristics. Where available, we have included imaging notes from the patient medical record.

The EEG were recorded on on a Natus Xltek EMU40EX system with built-in high and low pass filters of 0.1 and 400 Hz. Further notch filtering was done after acquisition. The data was recorded at 256 Hz using the 10–20 common reference and was resampled to 200 Hz to be consistent with the JHH dataset.

All EEG data was collected during routine clinical care and was anonymized prior to analysis under an approved IRB protocol at each participating site (JHH and UWM). Following [48], we bandpass each recording between 0.5 to 30 Hz and remove high intensity artifacts by thresholding each recording at two standard deviations from its mean value. The EEG signals were then normalized to have mean zero and variance one. The EEG signals were then normalized to have mean zero and variance one. One second non-overlapping windows were extracted for input to the models. For efficiency, we train the JHH detection models on EEG data containing 2 minutes of pre- and post-seizure activity; however, we evaluate them on the full 20-minute recordings.

#### Detection performance

Table 2 reports the detection results on the JHH dataset. We observe that the SZTrack and CNN-BLSTM exhibit nearly comparable performance at the window level, with AU-ROCs of 0.895 and 0.899, respectively. This comparison with the highly-optimized multichannel CNN-BLSTM show the ability of our simpler SZTrack model to achieve state-of-the-art seizure detection performance while preserving channel-wise information. Beyond these two models, the CNN-2D achieves the next-best overall performance, as highlighted by the AU-ROC and AU-PRC measures. This performance may be due to the relatively simple architecture, which can leverage the spectral input information. The remaining baselines are clustered together, with the ablated No-BLSTM and the two GCN models (Deep and Shallow) outperforming the recently proposed TGCN and Wei-CNN methods. We note that the TGCN and Wei-CNN baselines rely on larger and more complex architectures, which may result in overfitting to the relatively modest JHH dataset.

**Table 2:** Performance on the JHH dataset. Seizure detection results on the JHH dataset. Window-level metrics are aggregated across one-second segments of the EEG. Seizure level results are calculated over the duration of the seizure interval.

	Window Level Results				Seizure Level Results		
Model	AU-ROC	AUC-PR	Sensitivity	Specificity	FPs/hr	Sensitivity	Latency (s)
SZTrack	0.895 ± 0.112	0.644 ± 0.281	0.593 ± 0.305	0.936 ± 0.065	13.05	0.865	12.35
CNN-BLSTM	0.899 ± 0.085	0.635 ± 0.241	0.590 ± 0.048	0.945 ± 0.048	16.46	0.919	10.84
No-BLSTM	0.797 ± 0.101	0.438 ± 0.224	0.518 ± 0.211	0.883 ± 0.092	8.17	0.894	21.07
TGCN	0.760 ± 0.124	0.485 ± 0.177	0.591 ± 0.183	0.821 ± 0.157	7.99	0.859	25.41
Deep-GCN	0.786 ± 0.0978	0.394 ± 0.218	0.485 ± 0.208	0.887 ± 0.078	8.05	0.823	23.56
Shallow-GCN	0.792 ± 0.097	0.412 ± 0.177	0.488 ± 0.183	0.892 ± 0.157	8.77	0.835	27.49
Wei-CNN	0.764 ± 0.156	0.488 ± 0.280	0.405 ± 0.279	0.921 ± 0.109	7.5	0.77	21.27
CNN-2D	0.824 ± 0.147	0.527 ± 0.247	0.470 ± 0.253	0.921 ± 0.109	10.2	0.84	25.39

Unlike the window-level results, where there is a clear ordering between the methods, the performance is mixed at the level of contiguous seizure detection. For example, SZTrack and the CNN-BLSTM achieve the lowest detection latency at the cost of higher false positive predictions per hour. In contrast, the No-BLSTM, TGCN and static GCNs (Deep and Shallow) make fewer false positive detections but have notably higher latency. In terms of sensitivity, the CNN-BLSTM performs the best at 0.919 with the No-BLSTM model a close second at 0.894. The two GCN models performed comparably with sensitivities of 0.823 (Deep) and 0.835 (Shallow), which is on par with SZTrack. Finally, the Wei-CNN method achieves considerably lower sensitivity than the others, perhaps due to the larger architecture and lack of temporal modeling. Taken together, our seizure detection experiment demonstrates the clinical utility of our simple channel-wise architecture and information fusion strategy.

To assess cross-site generalization, Table 3 reports the seizure detection performance of the JHH models when evaluted on the UWM dataset. In this case, we recalibrate the detection threshold for each of the JHH models (obtained via LOPO-CV) on the UWM dataset, but we do not retrain the model parameters on the new data. Consequently, there is a performance decline across all models when translated from the JHH adult cohort to the UWM pediatric population. Nonetheless, we observe the same general trends. Namely, SZTrack shows comparable performance to the strictly detection based CNN-BLSTM model at the window level with AU-ROCs of 0.813 and 0.857, respectively. At the seizure level, SZTrack exhibits higher sensitivity (0.639) at the cost of more FPs/hr (14.05) when compared with the CNN-BLSTM, which had sensitivity and false positive rate of 0.523 and 2.83. Surprisingly, the Wei-CNN shows high generalization performance, with an AU-ROC of 0.849, exceeding its AU-ROC of 0.764 in the JHH dataset. This might be linked to the fact that the Wei-CNN was optimized for detection on the publicly available Children’s Hospital of Boston (CHB) dataset [8], which also contains pediatric patients. Similarly, the CNN-2D shows a high level of generalization stability across datasets, perhaps indicating the robustness of spectral information.

**Table 3:** Generalization detection results on the UWM dataset. Seizure detection performance when applying the JHH models to data from UWM. We ran a LOPO-CV on UWM to calibrate the seizure versus baseline detection threshold. However, we did not retrain the neural network weights.

	Window Level Results				Seizure Level Results		
Model	AU-ROC	AUC-PR	Sensitivity	Specificity	FPs/hr	Sensitivity	Latency (s)
SZTrack	0.813 ± 0.164	0.380 ± 0.301	0.427 ± 0.288	0.950 ± 0.060	14.05	0.639	5.48
CNN-BLSTM	0.857 ± 0.116	0.393 ± 0.298	0.329 ± 0.291	0.954 ± 0.083	2.83	0.523	12.28
No-BLSTM	0.724 ± 0.213	0.350 ± 0.312	0.287 ± 0.273	0.961 ± 0.073	7.48	0.517	12.65
TGCN	0.691 ± 0.205	0.257 ± 0.240	0.270 ± 0.228	0.894 ± 0.107	15.14	0.613	14.85
Deep-GCN	0.679 ± 0.219	0.285 ± 0.293	0.211 ± 0.206	0.958 ± 0.063	10.57	0.528	15.66
Shallow-GCN	0.699 ± 0.214	0.302 ± 0.299	0.245 ± 0.227	0.962 ± 0.063	8.77	0.557	16.39
Wei-CNN	0.849 ± 0.126	0.406 ± 0.291	0.536 ± 0.332	0.900 ± 0.145	11.11	0.701	5.87
CNN-2D	0.782 ± 0.157	0.382 ± 0.272	0.442 ± 0.221	0.956 ± 0.056	10.21	0.728	13.89

**Fig 4:**
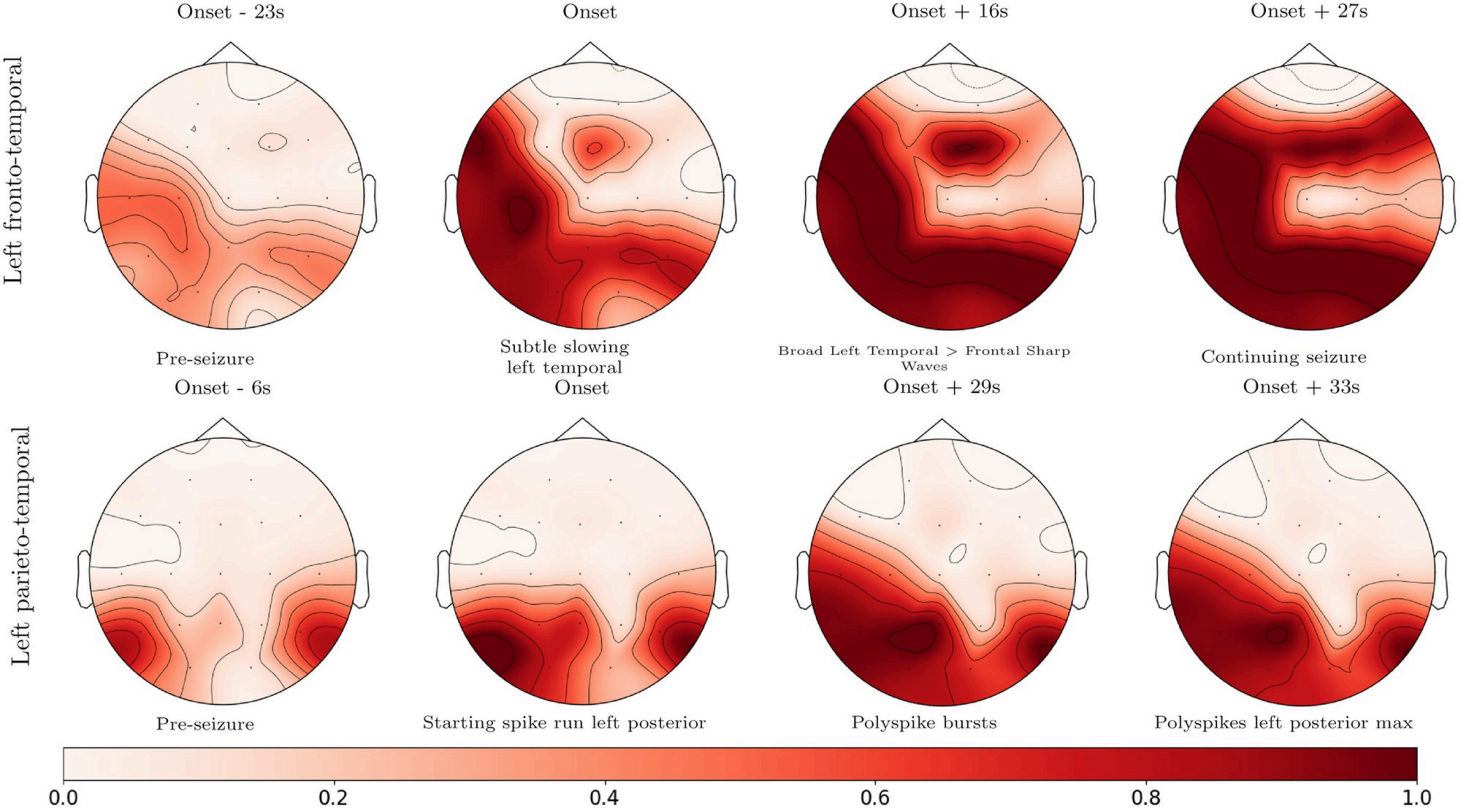
Seizure activity tracking. Seizure activity tracking in two JHH patients. Clinical SOZ annotations are given for each patient. Where clinical annotations are provided, images show seizure activity tracking corresponding to annotation times.

Fig 4 illustrates the seizure tracking output $P({\hat{Y}}_{i}\left[t\right]|X)$ by SZTrack for two patients from the JHH dataset, as superimposed in red on a topographic scalp plot. These recordings contain annotations of seizure spreading created by epileptologists during clinical workup. Seizure activity maps are provided at the time of annotation to show concordance between annotated seizure activity and SZTrack predictive outputs. As seen, the seizure activity *automatically learned* by SZTrack from the EEG data shows strong agreement with clinically observed spreading patterns during the seizure. We emphasize that due to our LOPO-CV training strategy, SZTrack had no *a priori* knowledge of these patients prior to generating the predictions in Fig 4. To the best of our knowledge, this is the first tacking result of its kind reported in the literature.

**Fig 5:**
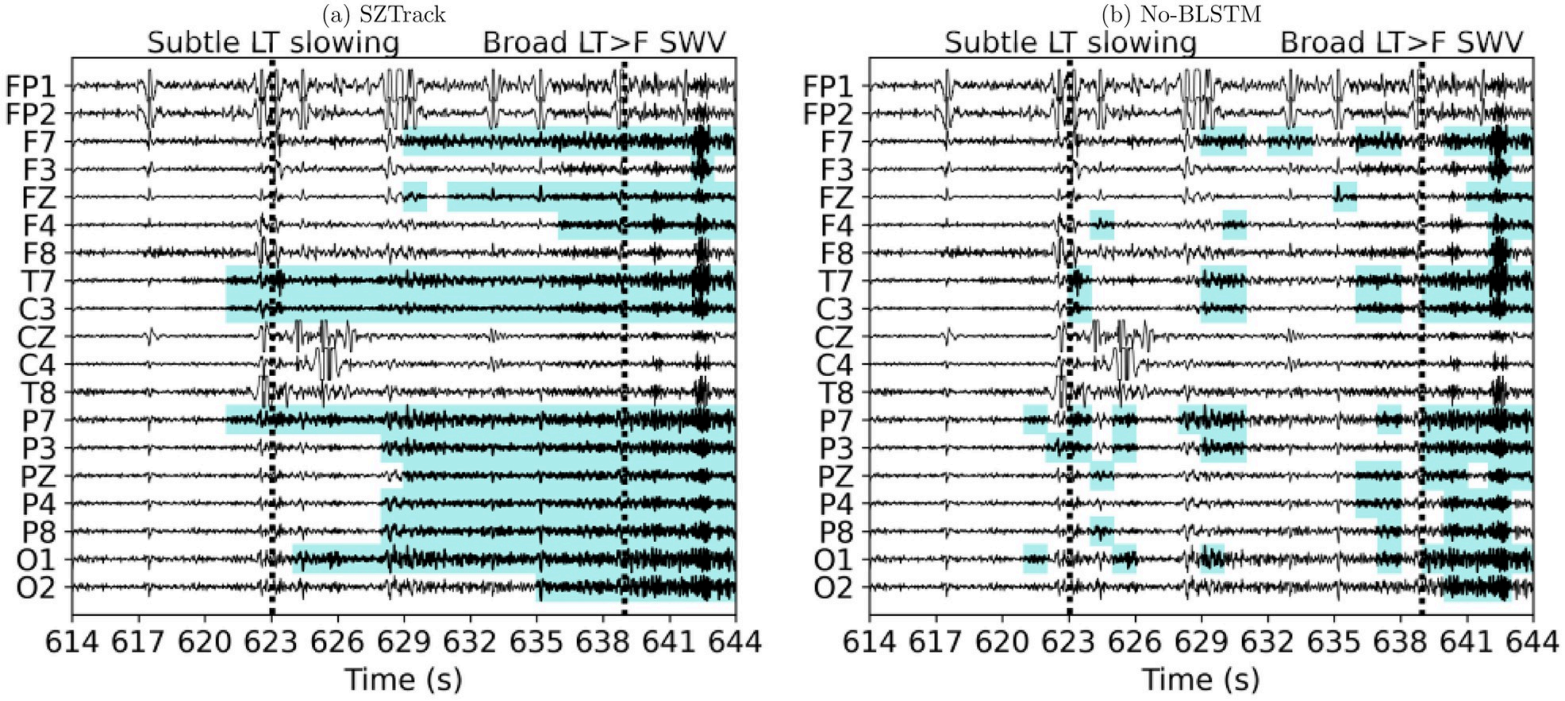
SZTrack and No-BLSTM output comparison. Channel-wise predictions for the fronto-temporal seizure shown on the top row of Fig 4 are superimposed on the EEG signal. In (a) SZTrack makes a confident prediction of seizure onset in the temporal channels which spreads to the parietal and frontal areas. In (b) No-BLSTM responds to isolated seizure activity at the onset but does not provide a temporally stable prediction.

Fig 5 illustrates the model predictions made by SZTrack and the No-BLSTM baseline for the fronto-temporal seizure shown on the top row of Fig 4. We have used the open-source EEG visualization software EPViz (https://engineering.jhu.edu/nsa/links/) to overlay the channel-wise predictions in blue on top of the EEG signals. As seen, SZTrack (a) predicts seizure activity originating in the temporal lobe channels T7 and P7. This prediction agrees with the clinically annotated onset information “subtle slowing left temporal” at 623 seconds in the EEG. Seizure activity quickly spreads to further involve left temporal, parietal, and left frontal electrode channels. Once again this prediction concurs with the clinical note of “Broad Left Temporal > Frontal Sharp Waves” at 639 seconds. In contrast, the No-BLSTM baseline correctly detects the left temporal onset but does not provide contiguous predictions. Consequently, it fails to capture the clinically observed spreading pattern.

#### Localization performance

**Fig 6:**
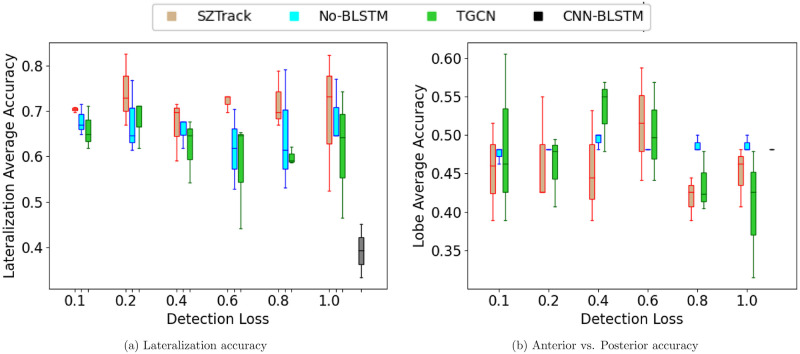
Localization sweep results. Average localization accuracy in JHH when varying the weight on the detection loss. Boxplots are shown for the SZTrack, No-BLSTM, and TGCN models. A horizontal dashed line shows performance for the CNN-BLSTM model.

Fig 6 illustrates the average lateralization and lobe classification performances in JHH dataset as the detection loss weight λ_*sz*_ is swept from zero to one. Accuracy results from each location class are averaged and boxplots from three separate runs are displayed for SZTrack, No-BLSTM, and the TGCN. The CNN-BLSTM baseline score is shown by the single gray boxplot, as this model was evaluated independently of seizure detection. The lateralization results are striking, as SZTrack uniformly outperforms the No-BLSTM and TGCN baselines, achieving its highest average accuracy of 0.826 at λ_*sz*_ = 0.6. The lobe identification task appears more difficult, as there is a universal decline in performance across all methods. In this case, the TGCN slightly outperforms SZTrack, achieving a maximum average lobe detection accuracy of 0.605 at λ_*sz*_ = 0.1 versus SZTrack’s 0.587 for λ_*sz*_ = 0.6. Nonetheless, SZTrack achieves robust detection and lateralization performance, thus illustrating itspotential clinical utility. We also note that this result is the first demonstration of end-to-end seizure localization from scalp EEG reported in the literature. With that said, further prospective analyses are required to evaluate the impact of SZTrack on the current clinical workflow.

**Fig 7:**
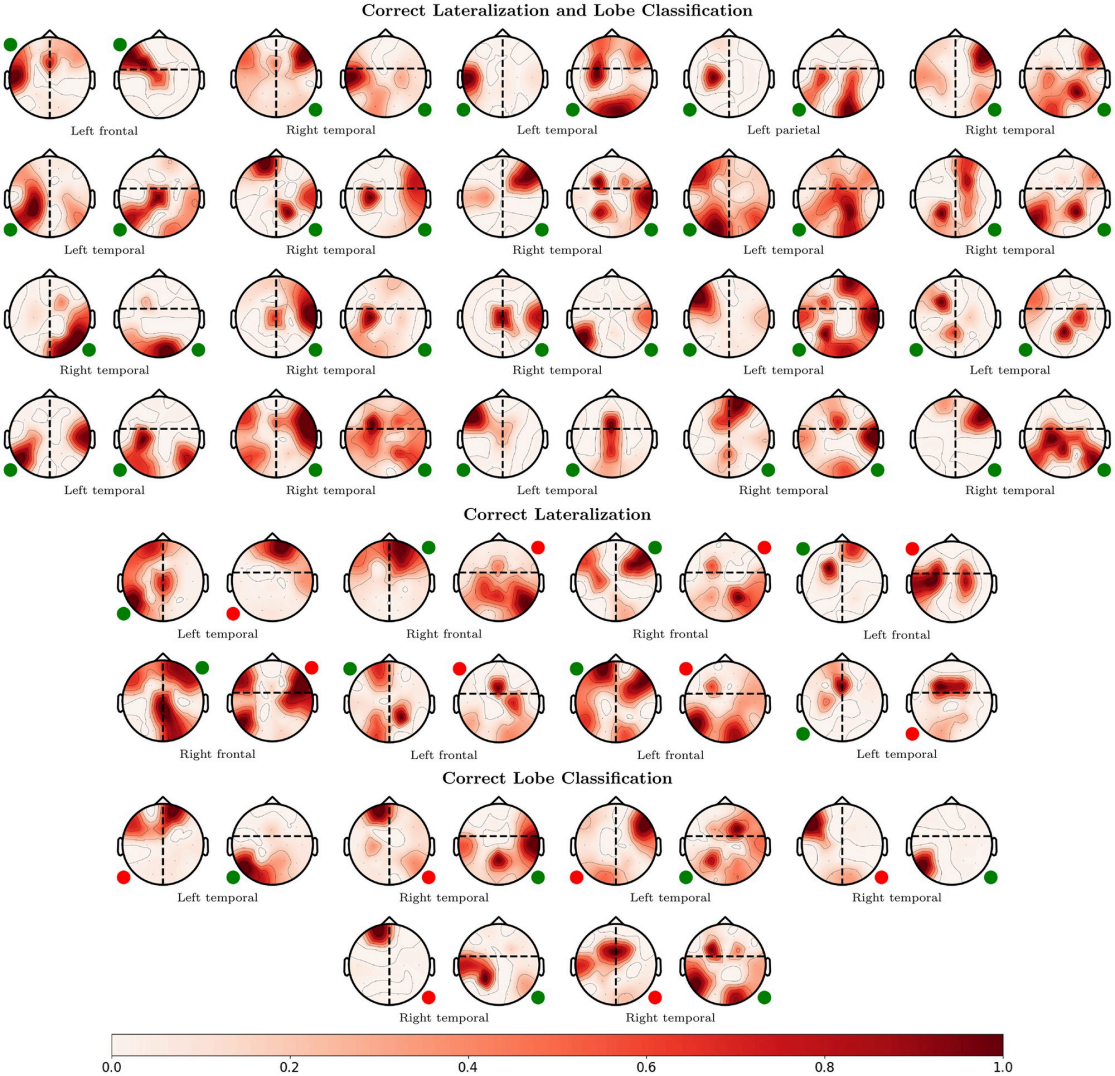
Localization results from the JHH dataset. Patient-wise lateralization and lobe classification for SZTrack in JHH. Predicted SOZ locations are superimposed on the head figure in red. The small circle indicates the coarse clinical SOZ annotation, where green indicates concordance with clinical annotations and red circle indicates disagreement. SZTrack correctly localizes both the hemisphere and lobe in 21 of 34 patients. In 12 of 34 patients, SZTrack correctly localizes either hemisphere or lobe; it misses completely in just one patient.

For a qualitative evaluation, Fig 7 illustrates the LOPO-CV localization results on the JHH dataset for a single hyperparameter setting (λ_*sz*_ = 0.2 for lateralization and λ_*sz*_ = 0.6 for lobe classification). The seizure onset maps, as denoted by *P*(**L**∣**X**), are shown superimposed on head plots in red. Lateralization and lobe images for each patient are displayed on the left and right, respectively, with the expert-determined SOZ provided below. For ease of comparison, we have added small circles to the corner associated with the clinical SOZ. A green circle indicates a concordance between SZTrack and clinical annotations while a red circle indicates disagreement. In 20 of 34 patients, SZTrack identifies both the correct hemisphere and lobe. SZTrack identifies the correct lobe or hemisphere in all of the 14 remaining patients.

**Fig 8:**
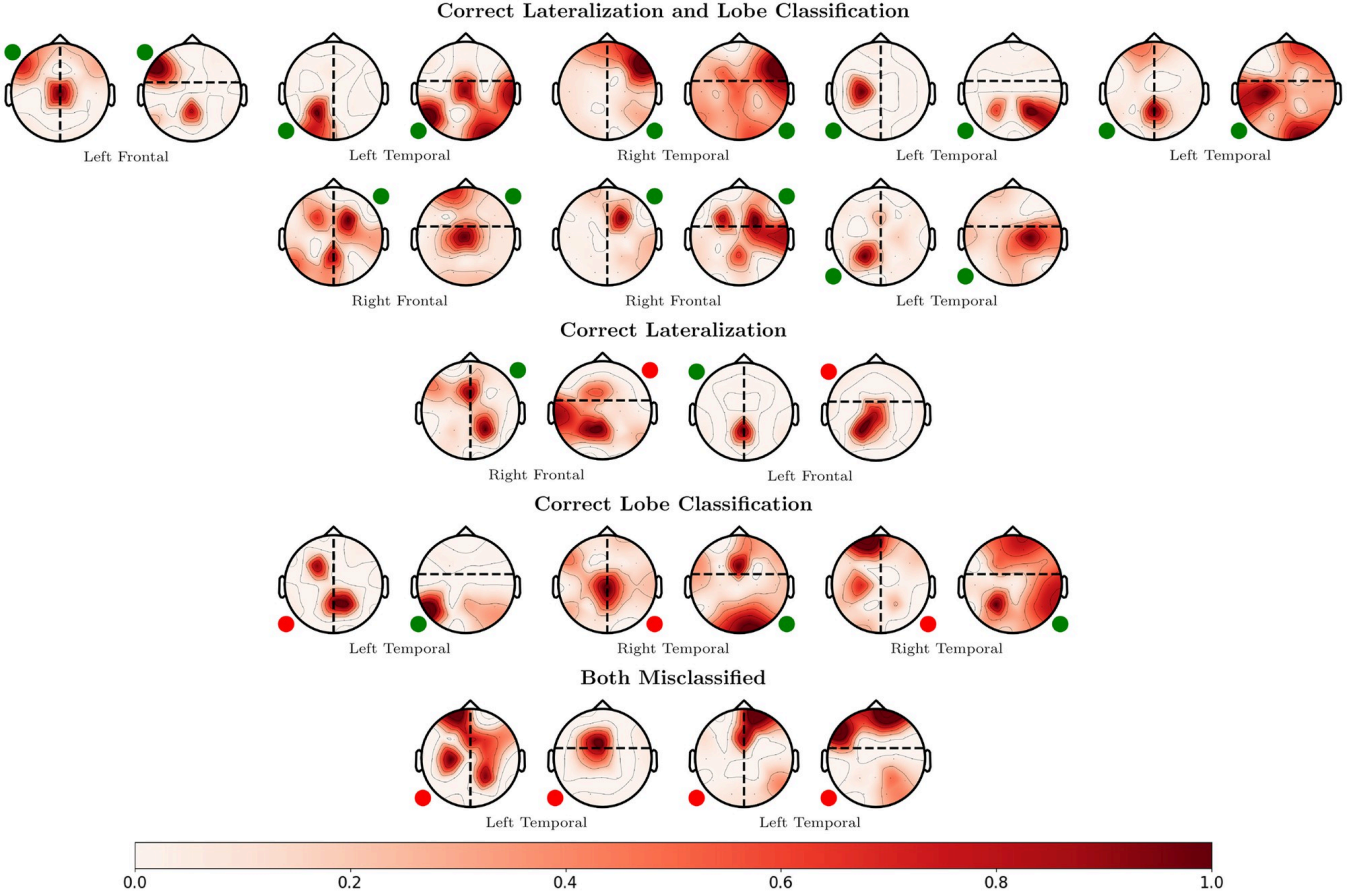
UWM dataset generalization results. Lateralization and lobe classification results when applying a SZTrack model trained on JHH to data from UWM. Predicted SOZ locations are shown superimposed on the head figure in red. The small circle indicates the coarse clinical SOZ annotation.

As a preliminary study of generalization, we selected a random LOPO-CV fold and applied the trained SZTrack model to the UWM data *with no fine tuning*. Fig 8 illustrates localization maps *P*(**L**∣**X**) for the hemisphere and lobe identification, as averaged across the seizure recordings for each patient. As seen, SZTrack correctly localizes both partitions in 8 of the 15 patients. In 5 of the patients, SZTrack correctly localizes either hemisphere or lobe. It misses completely in only 2 of the 15 patients. This result suggests that our SZTrack architecture is capturing salient information regarding seizure onset location that generalizes across different epilepsy cohorts.

## Discussion

SZTrack introduces a channel-wise architecture that consists of a CNN encoder, operating on one second windows of the EEG signal, followed by a BLSTM to capture both short- and long-term temporal dependencies. While the SZTrack architecture analyzes each EEG channel individually, our novel training strategy allows the network to learn and predict seizure activity based on the multichannel data. These predictions show high concordance with clinician determination of the seizure onset and offset. In addition, we have demonstrated the first end-to-end *seizure localization* results based on multichannel scalp EEG. Excitingly, SZTrack is able to *track* spatiotemporal seizure activity at higher resolution than the clinician annotations used for training.

In terms of seizure detection, SZTrack performs comparably to the benchmark CNN-BLSTM architecture. This result demonstrates that our max-pooling aggregation strategy can account for multichannel phenomena to a similar extent as a larger cross-channel architecture. Going one step further, Figs 4 and 5 demonstrate that SZTrack can learn channel-level seizure onset and propagation, a task that is impossible for the CNN-BLSTM. Specifically, it is not possible to disentangle which EEG channel(s) are driving the CNN-BLSTM prediction at any given time. In Fig 5 the added benefit of the LSTM for temporal seizure activity in the SZTrack architecture can be seen when comparing to model outputs provided by the No-BLSTM baseline. While the No-BLSTM baseline identifies some seizure activity near the annotated onset, it does not produce temporally contiguous seizure predictions after this onset. With the added LSTM layer, SZTrack correctly infers the start and continuing evolution of seizure activity. We stress that the tracking patterns are learned solely from annotations of the seizure onset and offset interval; our dataset does not contain fine-grained information at the level of individual channels.

When compared with GCN baselines, SZTrack exhibits higher seizure detection performance. These GCN models encode hypothesized network structures of the brain directly in their architectures in an attempt to capture “biologically informed” relationships in the data. Empirically, we observe that this approach does not directly lead to increases in detection performance, as SZTrack outperforms these graph based approaches. In fact, the results in Table 2 suggest that it may be more valuable to incorporate multichannel information during training (e.g., our max-pooling strategy) rather than directly into the network architecture. This is particularly true if there is a mismatch between the assumed graph structure and the actual EEG data.

In addition to detection efficacy, we make a first attempt to perform and validate end-to-end seizure onset localization from scalp EEG. SZTrack outperforms the ablated No-BLSTM, demonstrating the necessity of modeling temporal dependencies for this challenging task. SZTrack also outperforms the TGCN, which relies on both a GCN layer for cross-electrode information sharing and 1D temporal convolutions for time-series modeling. Similar to the detection task, perhaps the lower TGCN performance reflects a mismatch between the assumed graph and the actual data dependencies. The CNN-BLSTM, trained only for seizure localization, shows the worst performance of all the models in lateralization, and near-chanceanterior vs. posterior detection accuracy. This model analyzes all EEG channels concurrently in its architecture, which likely blurs subtle differences indicative of the seizure onset location.

We note a general decrease inanterior vs. posterior classification for all models. We hypothesize that this decrease is partially attributed to class imbalance. Most patients in our datasets have temporal lobe onsets, which greatly reduces the number of examples to learn patterns associated with other onset locations. Interestingly, in Fig 7 we note that in roughly 6 of 8 cases where SZTrack fails to correctly localize the SOZ in the anterior vs. posterior classification task, during the lateralization taskSZTrack places the mode of its SOZ probability in the correct anterior or posterior head region. This indicates that without the confounding effects of the dataset imbalance, SZTrack may learn to correctly classify anterior or posterior head regions even without being explicitly trained to do so.

The coarse division of onset zones intoanterior versusposterior may also contribute to the performance decrease, as it does not accommodate seizures originating at the border of this division or complex multi-focal cases. Particularly, we note that electrodes F7 and F8 may be involved in both frontal and temporal lobe onsets [49], while our division scheme necessitates that these electrodes be placed in only one head region. Future work will consider a more comprehensive channel grouping strategy that allows for soft assignments and overlapping classes. In theanterior vs. posterior classification task, we noted more variation in performance across models as the hyperparameter λ_*sz*_ is swept across its range. While the CNN-BLSTM drastically under-performed in the lateralization task, we note that its performance inanterior vs. posterior classification is on par with SZTrack and the other two baselines, likely due to the general difficulty of this task.

When applied to a generalization dataset, SZTrack shows robustness without the need for retraining. In the detection task, SZTrack maintains a stable AU-ROC across the JHH and UWM datasets. In the localization feasibility study, SZTrack models trained in the JHH dataset correctly identify the SOZ to the hemisphere andanterior vs. posterior level consistent with models trained and tested in the original dataset. These results demonstrate the robustness of SZTrack to real-world changes in clinical condition.

While we have demonstrated theretrospective clinical utility of SZTrack in detecting, tracking, and localizing seizure activity intwo separate scalp EEGdatasets, we note that our method has several limitations. While SZTrack achieves high seizure detection, the CNN-BLSTM surpasses SZTrack in performance in the original JHH dataset and UWM generalization dataset. Future work will incorporate a multi-channel component to SZTrack, similar to the CNN-BLSTM, to leverage cross-channel dependencies when making a prediction. Another issue is the relatively pooranterior vs. posterior classification performance. As described above, some issues inanterior vs. posterior classification may stem from our choice of dividing boundary betweenanterior and posterior SOZs. Specifically, onsets that occur near the boundary (e.g., fronto-temporal SOZ) are likely difficult for SZTrack to disambiguate. This scenario motivates a finer evaluation and training strategy foranterior vs. posterior prediction. In the future, we will explore data augmentation and aggregation techniques to increase the amount of extra-temporal seizure data for model training. Finally, validation of SZTrack in clinical settings is required to confirm its efficacy in prospective applications in the long term monitoring settings.

## Conclusion

We have introduced SZTrack, a novel deep neural network architecture for seizure activity tracking in multichannel EEG. Through cross electrode parameter sharing and novel predictive output aggregation, SZTrack achieves comparable seizure detection performance as deep models that use GCNs for direct information sharing between EEG electrodes. In addition, our aggregation techniques allow SZTrack to predict electrode level seizure activity from coarser clinical annotations. We also evaluate SZTrack on the difficult task of seizure localization, where it achieves high hemisphere and above-chanceanterior vs. posterior region classification accuracy. The localization performance also generalizes across sites with no fine tuning. SZTrack represents the first end-to-end neural network for seizure tracking, detection, and localization, establishing an important benchmark for the field.

## Supporting information

S1 Appendix JHH dataset demographics. Patient information including sex, age, seizure focus localiztion, and other relevant notes are given for patients in the JHH dataset.
(PDF)

S2 Appendix UWM dataset demographics. Patient information including sex, age, seizure focus localiztion, and other relevant notes are given for patients in the UWM dataset.
(PDF)

S1 Movie Seizure activity tracking for Fig 4 top row. SZTrack outputs for the evolving seizure shown in the top row of Fig 4 are displayed as a movie.
(MP4)

S2 Movie Seizure activity tracking for Fig 4 bottom row. SZTrack outputs for the evolving seizure shown in the bottom row of Fig 4 are displayed as a movie.
(MP4)

## References

[1] Fisher RS, Acevedo C, Arzimanoglou A, Bogacz A, Cross JH, Elger CE, et al. ILAE official report: a practical clinical definition of epilepsy. Epilepsia. 2014;55(4):475–482. doi: 10.1111/epi.12550 24730690

[2] French JA. Refractory epilepsy: clinical overview. Epilepsia. 2007;48:3–7. doi: 10.1111/j.1528-1167.2007.00992.x 17316406

[3] Rosenow F, Lüders H. Presurgical evaluation of epilepsy. Brain. 2001;124(9):1683–1700. doi: 10.1093/brain/124.9.1683 11522572

[4] van Donselaar CA, Schimsheimer RJ, Geerts AT, Declerck AC. Value of the electroencephalogram in adult patients with untreated idiopathic first seizures. Archives of neurology. 1992;49(3):231–237. doi: 10.1001/archneur.1992.00530270045017 1536624

[5] Osorio I, Zaveri H, Frei M, Arthurs S. Epilepsy: The intersection of neurosciences, biology, mathematics, engineering, and physics; 2016.

[6] Wu D, Wang Z, Jiang L, Dong F, Wu X, Wang S, et al. Automatic Epileptic Seizures Joint Detection Algorithm Based on Improved Multi-Domain Feature of cEEG and Spike Feature of aEEG. IEEE Access. 2019;7:41551–41564. doi: 10.1109/ACCESS.2019.2904949

[7] Shoeb A, Kharbouch A, Soegaard J, Schachter S, Guttag J. A Machine-Learning Algorithm for Detecting Seizure Termination in Scalp EEG. Epilepsy and Behavior. 2011;22:S36–S43. doi: 10.1016/j.yebeh.2011.08.040 22078516

[8] Shoeb AH, Guttag JV. Application of Machine Learning to Epileptic Seizure Detection. In: ICML: International Conference on Machine Learning; 2010. p. 975–982.

[9] Acharya UR, Molinari F, Sree SV, Chattopadhyay S, Ng KH, Suri JS. Automated Diagnosis of Epileptic EEG using Entropies. Biomedical Signal Processing and Control. 2012;7:401–408. doi: 10.1016/j.bspc.2011.07.007

[10] Guler NF, Ubeyli ED, Guler I. Recurrent Neural Networks Employing Lyapunov Exponents for EEG Signals Classification. Expert Systems with Applications. 2005;29(3):501–514. doi: 10.1016/j.eswa.2005.04.011

[11] Zandi AS, Javidan M, Dumont GA, Tafreshi R. Automated Real-Time Epileptic Seizure Detection in Scalp EEG Recordings Using an Algorithm Based on Wavelet Packet Transform. IEEE Transactions on Biomedical Engineering. 2010;57(7):1639–1651. doi: 10.1109/TBME.2010.2046417 20659825

[12] Zorlu M, Chuang D, Kettani H, Zarnegar R. F84. Sensitivity of Persyst Seizure Detection for Different Electrographic Seizure Patterns in Patients with Status Epilepticus. Clinical Neurophysiology. 2018;129:e98. doi: 10.1016/j.clinph.2018.04.247

[13] Bandarabadi M, Teixeira CA, Rasekhi J, Dourado A. Epileptic Seizure Prediction using Relative Spectral Power Features. Clinical Neurophysiology. 2015;126(2):237–248. doi: 10.1016/j.clinph.2014.05.022 24969376

[14] Alickovic E, Kevric J, Subasi A. Performance evaluation of empirical mode decomposition, discrete wavelet transform, and wavelet packed decomposition for automated epileptic seizure detection and prediction. Biomedical signal processing and control. 2018;39:94–102. doi: 10.1016/j.bspc.2017.07.022

[15] Gao Y, Gao B, Chen Q, Liu J, Zhang Y. Deep Convolutional Neural Network-Based Epileptic Electroencephalogram (EEG) Signal Classification. Frontiers in Neurology. 2020;11. doi: 10.3389/fneur.2020.00375 32528398 PMC7257380

[16] Yuan Y, Xun G, Jia K, Zhang A. A Multi-View Deep Learning Framework for EEG Seizure Detection. IEEE journal of biomedical and health informatics. 2018;23(1):83–94. doi: 10.1109/JBHI.2018.2871678 30624207

[17] Wei Z, Zou J, Zhang J, Xu J. Automatic epileptic EEG detection using convolutional neural network with improvements in time-domain. Biomedical Signal Processing and Control. 2019;53:101551. doi: 10.1016/j.bspc.2019.04.028

[18] Zou L, Liu X, Jiang A, Zhousp X. Epileptic Seizure Detection Using Deep Convolutional Network. In: 2018 IEEE 23rd International Conference on Digital Signal Processing (DSP). IEEE; 2018. p. 1–4.

[19] O’Shea A, Lightbody G, Boylan G, Temko A. Neonatal seizure detection from raw multi-channel EEG using a fully convolutional architecture. Neural Networks. 2020;123:12–25. doi: 10.1016/j.neunet.2019.11.023 31821947

[20] Vidyaratne L, Glandon A, Alam M, Iftekharuddin KM. Deep recurrent neural network for seizure detection. In: 2016 International Joint Conference on Neural Networks (IJCNN). IEEE; 2016. p. 1202–1207.

[21] Hu X, Yuan S, Xu F, Leng Y, Yuan K, Yuan Q. Scalp EEG classification using deep Bi-LSTM network for seizure detection. Computers in Biology and Medicine. 2020;124:103919. doi: 10.1016/j.compbiomed.2020.103919 32771673

[22] Liang W, Pei H, Cai Q, Wang Y. Scalp eeg epileptogenic zone recognition and localization based on long-term recurrent convolutional network. Neurocomputing. 2020;396:569–576. doi: 10.1016/j.neucom.2018.10.108

[23] Craley J, Johnson E, Jouny C, Venkataraman A. Automated inter-patient seizure detection using multichannel Convolutional and Recurrent Neural Networks. Biomedical Signal Processing and Control. 2021;64:102360. doi: 10.1016/j.bspc.2020.102360

[24] Zhou J, Cui G, Zhang Z, Yang C, Liu Z, Wang L, et al. Graph neural networks: A review of methods and applications. arXiv preprint arXiv:181208434. 2018;.

[25] Such FP, Sah S, Dominguez MA, Pillai S, Zhang C, Michael A, et al. Robust spatial filtering with graph convolutional neural networks. IEEE Journal of Selected Topics in Signal Processing. 2017;11(6):884–896. doi: 10.1109/JSTSP.2017.2726981

[26] Wagh N, Varatharajah Y. EEG-GCNN: Augmenting Electroencephalogram-based Neurological Disease Diagnosis using a Domain-guided Graph Convolutional Neural Network. In: Machine Learning for Health. PMLR; 2020. p. 367–378.

[27] Covert I, Krishnan B, Najm I, Zhan J, Shore M, Hixson J, et al. Temporal graph convolutional networks for automatic seizure detection. arXiv preprint arXiv:190501375. 2019;.

[28] Lian Q, Qi Y, Pan G, Wang Y. Learning graph in graph convolutional neural networks for robust seizure prediction. Journal of neural engineering. 2020;17(3):035004. doi: 10.1088/1741-2552/ab909d 32375134

[29] Yin Y, Zheng X, Hu B, Zhang Y, Cui X. EEG emotion recognition using fusion model of graph convolutional neural networks and LSTM. Applied Soft Computing. 2021;100:106954. doi: 10.1016/j.asoc.2020.106954

[30] Li X, Qian B, Wei J, Li A, Liu X, Zheng Q. Classify EEG and reveal latent graph structure with spatio-temporal graph convolutional neural network. In: 2019 IEEE International Conference on Data Mining (ICDM). IEEE; 2019. p. 389–398.

[31] Yan S, Xiong Y, Lin D. Spatial temporal graph convolutional networks for skeleton-based action recognition. In: Proceedings of the AAAI conference on artificial intelligence. vol. 32; 2018.

[32] Fuchs M, Wagner M, Kohler T, Wischmann HA. Linear and Nonlinear Current Density Reconstructions. J Clinical Neurophysiology. 1999;16:267–295. doi: 10.1097/00004691-199905000-00006 10426408

[33] Fuchs M, Ford MR, Sands S, Lew HL. Overview of Dipole Source Localization. Phys Med Rehabil Clin N Am. 2004;15:251–262. doi: 10.1016/S1047-9651(03)00126-8 15029908

[34] Wang JZ, Williamson SJ, Kaufman L. Magnetic Source Imaging Based on the Minimum-Norm Least-Squares Inverse. Brain Topography. 1993;5:365–371. doi: 10.1007/BF01128692 8357709

[35] Grech R, Cassar T, Muscat J, Camilleri KP, Fabri SG, Zervakis M, et al. Review on Solving the Inverse Problem in EEG Source Analysis. Journal of NeuroEngineering and Rehabilitation. 2008;5(1):25. doi: 10.1186/1743-0003-5-25 18990257 PMC2605581

[36] Gorodnitsky IF, Rao BD. Sparse Signal Reconstruction from Limited Data Using FOCUSS: A Re-weighted Minimum Norm Algorithm. IEEE Transactions on Signal Processing. 1997;45. doi: 10.1109/78.558475

[37] Cuffin BN. EEG Localization Accuracy Improvements using Realistically Shaped Head Models. IEEE Trans Biomedical Engineering. 1996;43:299–393. doi: 10.1109/10.486287 8682542

[38] Crouzeix A, Yvert B, Bertrand O, Pernier J. An Evaluation of Dipole Reconstruction Accuracy with Spherical and Realistic Head Models in MEG. Clinical Neurophysiology. 1999;110:2176–2188. doi: 10.1016/S1388-2457(99)00174-1 10616124

[39] Foged MT, Martens T, Pinborg LH, Hamrouni N, Litman M, Rubboli G, et al. Diagnostic added value of electrical source imaging in presurgical evaluation of patients with epilepsy: a prospective study. Clinical Neurophysiology. 2020;131(1):324–329. doi: 10.1016/j.clinph.2019.07.031 31466846

[40] Maas AL, Hannun AY, Ng AY, et al. Rectifier nonlinearities improve neural network acoustic models. In: Proc. icml. vol. 30. Citeseer; 2013. p. 3.

[41] Ioffe S, Szegedy C. Batch normalization: Accelerating deep network training by reducing internal covariate shift. In: International conference on machine learning. PMLR; 2015. p. 448–456.

[42] He K, Zhang X, Ren S, Sun J. Deep residual learning for image recognition. In: Proceedings of the IEEE conference on computer vision and pattern recognition; 2016. p. 770–778.

[43] Brazier MA. Spread of seizure discharges in epilepsy: anatomical and electrophysiological considerations. Experimental neurology. 1972;36(2):263–272. doi: 10.1016/0014-4886(72)90022-2 4559716

[44] Grinenko O, Li J, Mosher JC, Wang IZ, Bulacio JC, Gonzalez-Martinez J, et al. A fingerprint of the epileptogenic zone in human epilepsies. Brain. 2018;141(1):117–131. doi: 10.1093/brain/awx306 29253102 PMC5837527

[45] Graves A, Fernández S, Schmidhuber J. Bidirectional LSTM networks for improved phoneme classification and recognition. In: International conference on artificial neural networks. Springer; 2005. p. 799–804.

[46] Craley J, Johnson E, Venkataraman A. A Spatio-Temporal Model of Seizure Propagation in Focal Epilepsy. IEEE Transactions on Medical Imaging. 2019; p. 1–1. 10.1109/TMI.2019.2950252 31675325

[47] Kipf TN, Welling M. Semi-supervised classification with graph convolutional networks. arXiv preprint arXiv:160902907. 2016;.

[48] Craley J, Johnson E, Venkataraman A. A novel method for epileptic seizure detection using coupled hidden markov models. In: Medical Image Computing and Computer-Assisted Intervention. Springer; 2018. p. 482–489.

[49] Marcuse LV, Fields MC, Yoo JJ. Rowan’s Primer of EEG E-Book. Elsevier Health Sciences; 2015.

