# Supplementary material for: Automated seizure activity tracking and onset zone localization from scalp EEG using deep neural networks
Source: PLoS One. 2022 Feb 28;17(2):e0264537. doi: 10.1371/journal.pone.0264537 (PMC8884583; doi:10.1371/journal.pone.0264537)
Supplement: S1 Appendix — Patient information including sex, age, seizure focus localiztion, and other relevant notes are given for patients in the JHH dataset. (PDF) [file pone.0264537.s001.pdf]

S1 Appendix: JHH Additional Demographics Table

Patient	Sex	Age	Localization Notes	Other notes	Ant./ Post.	Right/ Left
1	M	50	Right temporal epilepsy, though hard to tell onsets	Cavernoma	P	R
2	F	32	Complex partial seizures with right frontocentral onset		A	R
3	F	52	Maximal over left temporal head region	MTS	P	L
4	F	25	Left frontocentral or left temporal, has periventricular nodular heterotopia	Periventricular heterotopia	A	L
5	M	77	Right temporal	Ovoid focus	P	R
6	M	52	Posterior left parietal	White matter disease	P	L
7	M	38	At first difficult lateralization, later thought to be left fronto-temporal	Possible MTS	A	L
8	F	46	Right temporal	History of stroke	P	R
9	M	39	Left frontal	Nonlesional brain MRI	A	L
10	F	41	Left frontal		A	L
11	F	29	Right fronto-temporal	Nonlesional MRI and PET	A	R
12	F	10	Left antero-temporal	Nonlesional brain MRI	P	L
13	M	45	Right or left temporal onset	Early evidence of MTS	P	R
14	F	12	Right temporal	MTS	P	R
15	M	20	Left temporal	Encephalocele	P	L
16	F	18	Right paracentral anterior frontal	FCD	A	R
17	F	45	Left mid/posterior temporal		P	L
18	M	74	Right temporal	MRI suggestive of MTS	P	R
19	F	33	Right temporal	MRI suggestive of MTS	P	R
20	F	19	Right temporal	MRI suggestive of MTS	P	R
21	M	51	Left temporal	MTS	P	L
22	M	49	Left temporal		P	L
23	F	22	Left posterior parietal-temporal region (P9, P7)	MRI suggestive of FCD	P	L
24	M	21	Right temporal		P	R
25	F	36	left anterior and mid temporal	Low grade glioma	P	L
26	F	25	Likely left temporal, however had prior R surgery, probably has bilateral seizures	MTS	P	L
27	M	17	Right anterior to mid temporal lobe	MTS	P	R
28	F	44	Right temporal (had right temporal tip encephalocele)	Encephalocele	P	R
29	M	22	Right temporal	MTS	P	R
30	F	41	Right temporal	MTS	P	R
31	F	35	Left temporal		P	L
32	M	56	Right fronto-temporal, thought to be temporal due to imaging	MRI consistent with MTS	P	R
33	M	6	Right temporal lobe		P	R
34	M	33	Bilateral temporal, thought to be left due to imaging	Abnormality, possibly cortical dysplasia or low grade glioma	P	L

Table 1: **JHH dataset demographics.** Patient information including sex, age, seizure focus localiztion, and other relevant notes are given for patients in the JHH dataset.
