# Supplementary material for: Automated seizure activity tracking and onset zone localization from scalp EEG using deep neural networks
Source: PLoS One. 2022 Feb 28;17(2):e0264537. doi: 10.1371/journal.pone.0264537 (PMC8884583; doi:10.1371/journal.pone.0264537)
Supplement: S2 Appendix — Patient information including sex, age, seizure focus localiztion, and other relevant notes are given for patients in the UWM dataset. (PDF) [file pone.0264537.s002.pdf]

S2 Appendix: UWM Additional Demographics Table

Patient	Sex	Age	Localization Notes	Other notes	Ant./ Post.	Right/ Left
A	M	14	Left anterior and medial parietal region, post central gyrus	FCD	P	L
B	M	8	Left posterior frontal and pre central gyrus, focal at C3	FCD	P	L
C	M	17	Right temporal lobe, focus at T4	FCD	P	R
D	M	15	Left anterior parietal region, max at C3	FCD	P	L
E	M	17	Left anterior temporal pole, focus at T1	Encephalocele	P	L
F	F	10	Left anterior frontal pole, focus at Fp1	FCD	A	L
G	F	13	Left frontal lobe	Gliosis, encephalomalacia	A	L
H	M	11	Right temporal lobe	Gliosis, MTS	P	R
J	F	17	Diffuse left onset, best developed in P3	Encephalitis	P	L
K	M	14	Left inferior frontal operculum and insula	Unknown	P	L
L	M	15	Right middle frontal region	FCD	A	R
M	F	11	Right middle frontal region, EEG max Fp2/F4	FCD, tuberous sclerosis	A	R
O	F	14	Hard to localize, Fz-Cz-Fp2-F4-C4 +/- P4?	FCD	P	R
Q	M	9	Right frontal maximum.	FCD	A	R
S	M	8	Focus at C3	FCD	P	L

Table 1: **UWM dataset demographics.** Patient information including sex, age, seizure focus localiztion, and other relevant notes are given for patients in the UWM dataset.
